# Computational metrology for materials

**DOI:** 10.1557/s43578-025-01651-2

**Published:** 2025-07-31

**Authors:** James Warren, Jake Read, Jonathan Seppala, Erik Strand, Neil Gershenfeld

**Affiliations:** 1 National Institute of Standards and Technology, Gaithersburg, USA; 2 MIT Center for Bits and Atoms, Cambridge, USA

**Keywords:** 3D printing, Additive manufacturing, Machine learning, Extrusion, Materials genome

## Abstract

Advanced materials hold great promise, but their adoption is impeded by the challenges of developing, characterizing, and modeling them, then of designing, processing, and producing something with them. Even if the results are open, the means to do each of these steps are typically proprietary and segregated. We show how principles of open-source software and hardware can be used to develop open instrumentation for materials science, so that a measurement can be accompanied by a complete computational description of how to reproduce it. And then we show how this approach can be extended to effectively measure predictive computational models rather than just model parameters. We refer to these interrelated concepts as “computational metrology.” These are illustrated with examples including a 3D printer that can do rheological characterization of unfamiliar and variable materials.

**Graphical abstract:**

A demonstration of computational metrology is shown through the development of a Rheoprinter (left) that combines off-the-shelf printer components with custom instrumentation. At right, a model made by the Rheoprinter to predict relative nozzle pressures as a function of material flow rate and nozzle temperature.

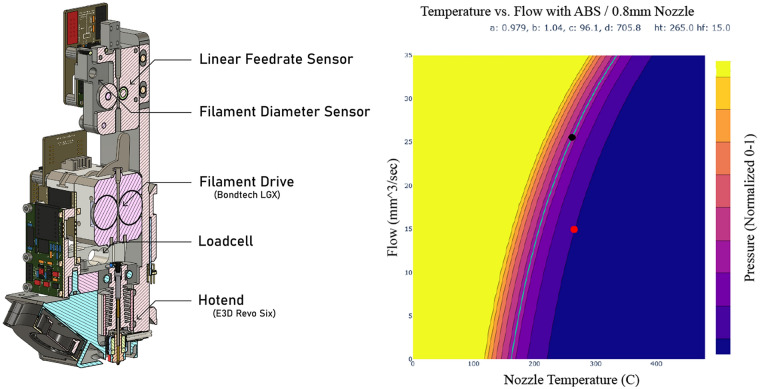

## Introduction

Accelerated methods for obtaining advanced materials has long been a goal of the research and development enterprise. To address this, The US Materials Genome Initiative (MGI) seeks to accelerate the discovery, design, development, and deployment of new materials at a fraction of the cost, through the creation of a materials innovation infrastructure. This experimental, computational, and data infrastructure lowers the barriers to materials design, and; thus, exists as a democratizing means to the end of accelerated materials R&D. In what follows, we will discuss how the concepts of open and computational metrology emerged in the MGI.

One of the primary motivations driving the establishment of the MGI was the need for a shift in how modeling and simulation were used as part of the materials R&D enterprise. It had long been the case that modeling was viewed as inferior to experimental approaches, and not as a crucial and equal partner. Building off prior materials modeling successes [1] the MGI sought to foster computational approaches that were tightly integrated with experiments, with a supporting data infrastructure to manage flows of information both within a lab, and with federated resources around the world.

The MGI’s focus on data and the potential of data-driven materials R&D [1] anticipated and then supported the subsequent rise of artificial intelligence-based models of materials synthesis and characterization. Now, thirteen years after the establishment of the MGI, all of these ideas are converging towards a conceptual framework we term “computational metrology.”

The focus of the MGI has been around mitigating the arduous process of designing and deploying a new material. There are numerous challenges that need to be overcome at each stage of the materials R&D process. There is a large loop from designing a material, to characterizing it, to modeling it, to designing with it, to processing and manipulating the material, to producing a finished product. In general each of these steps can be quite time consuming and expensive, employing proprietary equipment and processes, which are often poorly integrated with other tools that make up the materials R&D enterprise. In addition, materials design requires specialized skills and training. Finally, materials R&D is continuously hindered by the lack of data. Often data is just not available, and when it is available it can be proprietary. The deployment of MGI-inspired tools will lower the barriers to efficiently exercising the large loop of materials development, while also reducing the required skill-sets, and allowing for the generation of material data on the fly, further democratizing access to these approaches. Indeed, as we will explore, one can go beyond just generating data for use in a model if one takes the more holistic view of computational metrology.

To measure something presumes the existence of a model that is assumed to be correct for the response of a system to a range of applied changes or probes. A linkage between measurement and model is typically done by controlling the variables besides the measurand to the highest degree possible, but this strategy, while time-tested, is not necessarily feasible. Instead, with the aid of computation, we can now solve highly complex models by indirectly inferring them from accessible observations. This is the heart of computational metrology.

Ultimately, this work is suggesting a shift in how measurements are described. In general, what is desired by most scientists and engineers is a model with predictive power that provides a pathway towards a desired objective (a stronger material, a more recyclable battery with double the lifetime,…). In many cases it will be expedient to bypass the idea of a single measured quantity, and jump directly to “measuring the model.” The combination of these ideas and the ability to share open-source specifications for instruments, and models of the measurement process, allows for wider democratization of materials research, and connects with a growing movement in open metrology.

## Open metrology

Metrology by definition requires openness, because measurements cannot be exchanged without agreement on their meaning. However, this openness may not extend to the means for making those measurements, which are typically performed using proprietary instrumentation. The instrument specifications and their traceability may be open, but not their implementation. This lack of transparency can be a barrier to their access, integration, and application.

An alternative approach is based on the use of open-source hardware [2]. This extends principles of open-source software to hardware. Along with freely sharing complete design specifications, these come with licenses specifying how they can be used and modified.

Open hardware is enabled by the spread in the availability and capabilities of rapid-prototyping tools, including physical fabrication, embedded electronics, and sensing and actuation. These are being combined to enable rapid prototyping of rapid-prototyping, for rapid machine building [3]. Those same elements can be used to create a range of open materials science instrumentation [4]. Examples include a rheometer [5], a Raman spectrometer [6], a fiber spectrometer [7], an optical microscope [8], an Atomic Force Microscope [9], a plastic scanner [10, 11], a liquid handling platform [12] used for synthesis of CdSe nanocrystals [13], and a PCR (polymerase chain reaction) thermal cycler [14]. In each case, these include a bill of materials, design files for additive and subtractive processes, schematics, and microcode, allowing not just experiments but the experimental apparatus to be reproduced.

One challenge for open hardware has to do with interchange formats; much of open software’s success is based on the availability of software design texts. Similar interchange formats for generalized data structures exist like Comma Separated Values (CSV), JavaScript Object Native (JSON), and Tom’s Own Markup Language (TOML), and designs and data using these formats are readily shared and collectively developed. Software designs are easily transformed into working code on almost any computing system by way of interpreters and compilers, despite a heterogeneity of processor and computer architectures.

Re-creating the mechatronic systems required to make real-world measurements requires a more heterogeneous set of design documents: embedded code that runs on physical devices is required, but in order to understand what a line of embedded code is doing in the physical world, we need also to see the circuit schematic where it lives, and also to read the datasheet provided by the microcontroller’s manufacturer. We also need datasheets and schematics of any other device on the circuit in question.

Beyond the circuit, we need physical representations of the rest of the instrument: an ADC or DAC (Analog to Digital Converter, and Digital to Analog Converter) might be connected to a coil, a heater, or a thermistor: how many windings are in the coil, at what diameter? Where is the coil positioned? These data can be encoded in physical design CAD (Computer Aided Design) software, but no standard interchange format exists that preserves 3D design intent, and the same is true of circuit designs. Both disciplines have standard output formats like Gerber and STEP (Standard for the Exchange of Product Data), and mature open-source editing tools, but neither has an interoperable editable interchange format.

Interoperable design formats across software and hardware would make it possible in hardware to do what is common in software: distributed development and improvement, global reproducibility, and application spanning reusability. Research articles in computer science are routinely published alongside working demo code—or better yet, alongside software modules that can be used by other researchers to further their own scientific efforts. The same is possible for hardware, though there is another challenge in this domain: not everyone has the same access to the supply chains and fabrication equipment that make it possible to reproduce designs [15]. Access to digital fabrication is pushing this boundary, allowing designs to be developed that are more readily fabricated using homogenous feedstocks, parametric CAD, and a common set of direct-write processes [16, 17].

**Figure 1:**
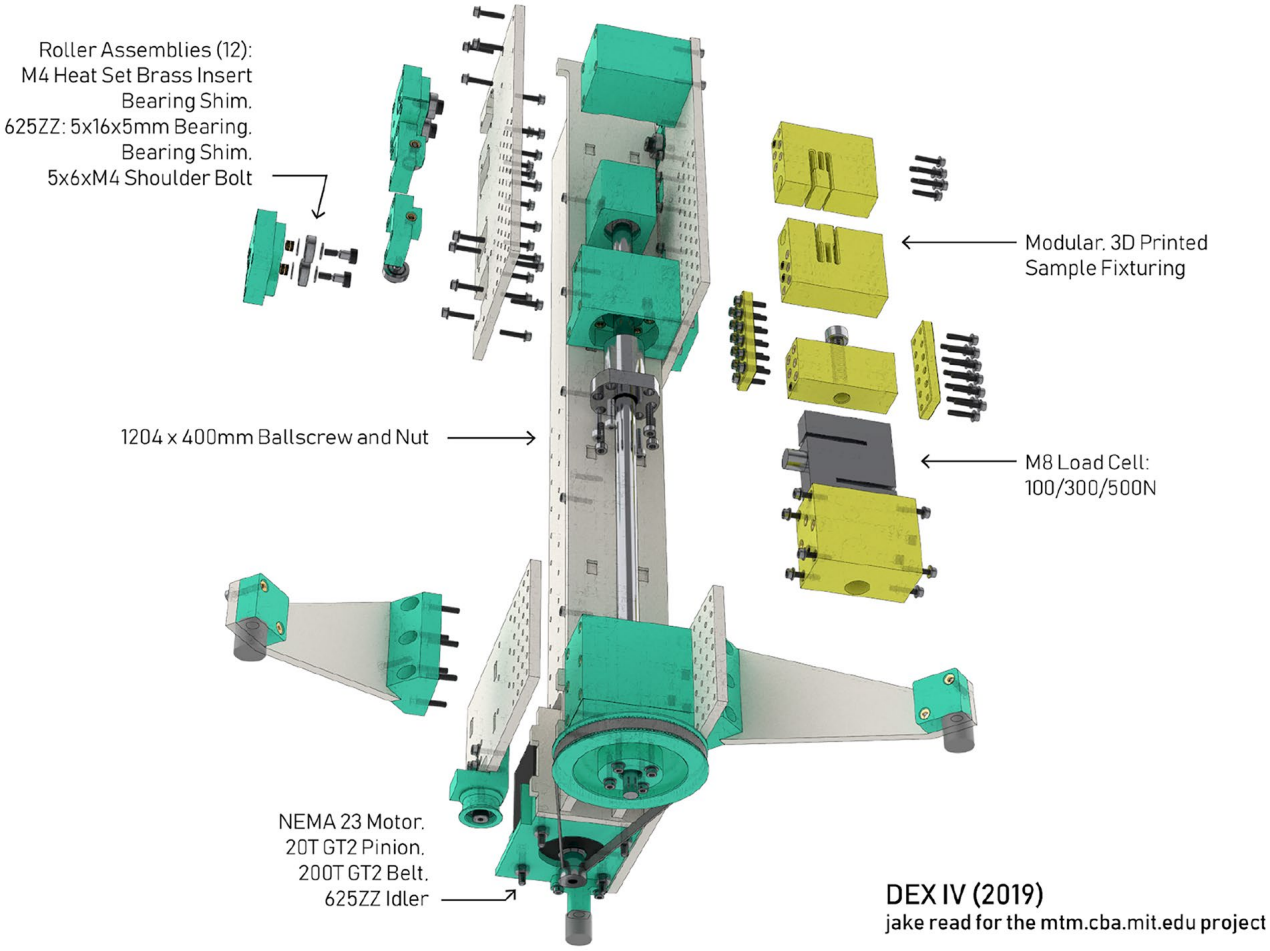
An exploded view of the Displacement Exercise (DEX), an open-source tensile testing machine [18].

Extending this effort, we developed an open, fabricatable tensile testing machine and published open-source files for the hardware (CAD) and electronics (developed on a breadboard with modules from other open-source hardware developers) and controllers (firmware and a browser-based interface) that we called the *displacement exercise* [18] (Fig. 1).

Like many of the other examples referenced earlier, DEX is a monolithic open-source project, *i.e.* it is a stand-alone device that is meant as a drop-in replacement for closed-source alternatives. While these kinds of projects can be valuable in many contexts (lowering barriers to access, and educating other would-be systems builders), their adoption has been small relative to their closed-source counterparts. Scholars who study the proliferation of open software would note a similarity to early efforts in that domain: to produce large end-user facing programs such as office suites that were meant to replace proprietary counterparts [19]. Open software’s contemporary success is not found in these types of programs, but in the countless libraries and packages that are available to other software developers. In this paradigm, functional building blocks are shared in a commons and re-used in many different application-specific projects [20]. Based on this insight, we have focused our successive efforts on the development of interoperable modular systems for low-level control of mechatronic devices [21, 22].

## Computational metrology

The goal of material measurements is not just obtaining a parameter, it's enabling an application. There are many intervening steps from a measurement, to a model, to its use in a process. In computational metrology, we seek to eliminate them by directly measuring a predictive model.

Computational metrology is based on the observation that end-use applications might not be able to directly access traditional model parameters, but we can instrument process technologies to characterize a range of process parameters that can serve as indirect observations of the underlying model. These can then be developed by reinforcement learning, and validated by testing their ability to generalize beyond the observations.

As an example of this indirect measurement technique, we can consider modeling the elastic and plastic deformation of a thermoplastic coupon. Traditionally, we would need to select a theoretical framework, such as linear elasticity for the elastic regime, and viscoelastic and/or viscoplastic theories for plastic deformation. Before we could apply these models, we would need to measure a variety of fundamental material properties, such as the Young’s modulus, shear modulus, Poisson’s ratio, yield strength, viscosity, and strain rate sensitivity, all of which may depend on temperature.

**Figure 2:**
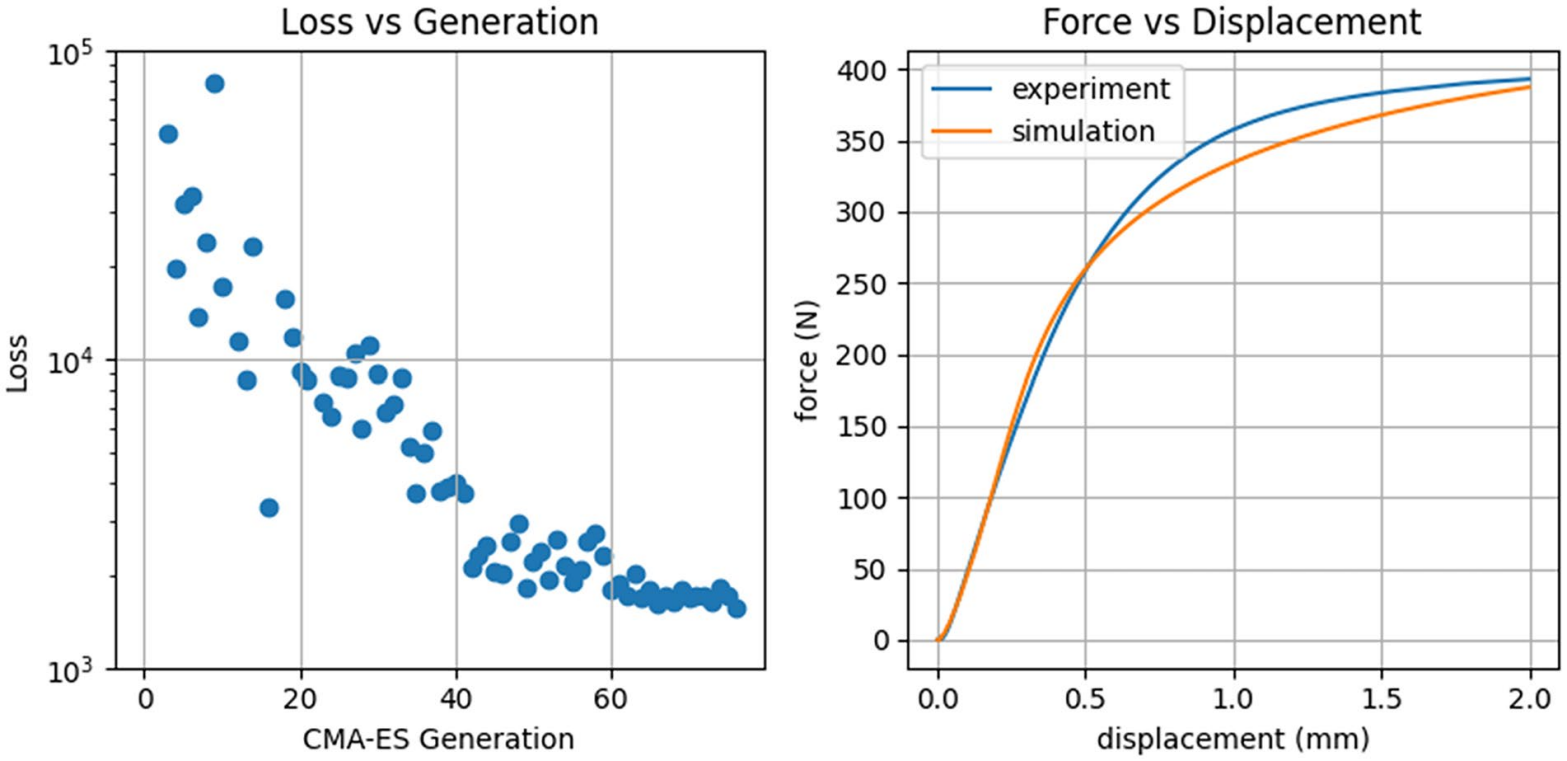
Optimization progress and resulting stress–strain curve for a MIPS model.

Alternatively, one may select a simple computational model with tunable generic parameters. This could be a neural network such as a physics informed neural network [23], or a particle model with a parametric force law such as memoryless isotropic point particles (MIPS) [24]. In either case, the model is used to directly predict whatever sensor measurements are available, and the generic parameters are optimized in order to make the simulated results match the physical results. For example, we may take as our objective function the force *vs* displacement curve measured by a machine such as DEX. A MIPS model can be fit to this data, and then directly used to predict deformation of a particular geometry under different loads. One simple model subsumes both the elastic and plastic regimes (Fig. 2).

This opens the door to process-specific measurement and prediction. By simulating each of the sensors onboard a machine (*e.g.* an FDM printer), as well as the function of the machine itself (*e.g.* extrusion), we can merge the metrological and control aspects of the entire system. This bypasses an entire suite of independent lab tests that determine traditional material properties. It can also function in real-time, so that process parameters can be adapted to new materials on the fly.

In addition, computational metrology provides a rigorous framework for the analysis and propagation of uncertainty in input variables with respect to the observational model, with validation through out-of-sample generalization as we've illustrated. As an inherently model-based approach to materials measurement and, ultimately, design, a systematic exploration of the influence of uncertainties in inputs and the sensitivity of the system to such variations can enable deeper insights into the measurement system and its limitations.

In manufacturing equipment we need models that are hybrids of the machine's dynamics and the materials’ properties, both of which have meaningful impact on how a machine would be optimally operated. For example an injection molding machine’s controller should have information about the maximum pressures and heat fluxes that can be generated by the machine, as well as the plastics’ rheological properties [25]. At the heart of this control paradigm is the increasing availability of software tools and libraries that make the development and deployment of optimization-based control easier [26, 27] and more general autograd packages [28].

**Figure 3:**
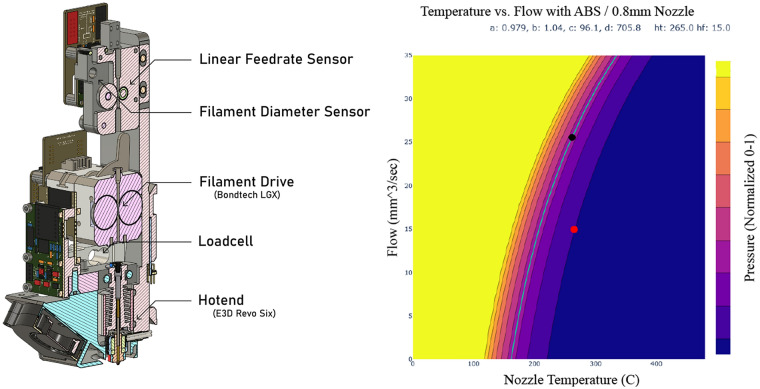
The Rheoprinter’s instrumented printhead (left) combines off-the-shelf printer components with custom instrumentation. At right, a model made by the Rheoprinter to predict relative nozzle pressures as a function of material flow rate and nozzle temperature.

To bring situated metrology online with process control and “machines learning” we developed the Rheoprinter, an FFF (Fused Filament Fabrication) printer that measures the materials it is using alongside its own dynamics (Fig. 3). The Rheoprinter adds to its extruder a load cell (for pressure measurement) and a filament sensor (to measure real feed rate into the hotend), based on Ref. [29]. These allow the machine to build machine-material models that predict pressures at given flowrates and temperatures. These models allow us to generate working process parameters in one shot, using a short experiment that takes about 15 min [30].

The system can work well with relatively low quality measurements because those measurements are normalized to the same system that the metrology is performed on. In the recent work, we have extended our system to account for dynamics, completing a controller for the machine that follows the design pattern laid out in the previous section. In this work, we use computer vision and the machine’s own controller to develop a model that describes flow rates given the system’s full dynamics (including filament compression). We couple this model with a kinematic model of the machine’s motion system, and use them together in an online optimizer (a Model Predictive Controller—MPC) to operate the machine. By doing so, we can bypass many of the feed-forward parameters that users normally need to set by hand.

This approach departs significantly from traditional rheological practices, where the viscoelastic response of polymers is determined under idealized conditions and used to develop constitutive models linking stress to strain. These models are then employed to make predictions or control process outcomes, aiming to capture the complete state of the system and the material's response to it. However, achieving this in real time is rarely feasible. Unlike purpose-built rheometers—costing between $10,000 and $100,000—that create isothermal environments, idealized flow fields, and often linear flows, with well-defined stress and strain conditions, the Rheoprinter captures critical aspects of rheology, such as stress and strain indices (load cell and filament encoder), but under nonisothermal and nonlinear conditions. Instead, its measurements reflect the process state, combining both the process conditions and material response. This approach significantly simplifies modeling by eliminating the need for a comprehensive multiphysics understanding of both the process and the material.

## Conclusion

With a focus on materials science, we've illustrated how metrology can migrate from proprietary to open-source tools, offering opportunities to perform faster (by reducing development time), better (by easing integration across devices and algorithms), and cheaper (by reducing costs over the bill of materials). The use of open hardware along with open software will aid reproducibility, by allowing not just the description of an experiment but also its implementation to be replicated. Realization of this vision will require a corresponding integration of open interchange formats, which today requires separate descriptions of mechanical design, production path planning, schematics, circuit boards, bills of material, microcode, interface protocols, and application code. It also requires a new paradigm for open-source systems integration to allow developers to easily build application-specific devices from modular building blocks.

Open metrology leads to computational metrology, by exposing internal degrees of freedom and allowing added instrumentation that is unavailable within the internals of a proprietary system. We've shown how this can be used to indirectly determine quantities that are not observed directly, and to effectively measure predictive computational models rather than just model parameters. Computational metrology is the embodiment of machine learning in machines.

## Data Availability

All data is available at https://gitlab.cba.mit.edu.

## References

[1] https://www.mgi.gov/sites/mgi/files/mgi_strategic_plan_-_dec_2014.pdf, 10.17226/12199

[2] https://www.oshwa.org

[3] https://mtm.cba.mit.edu

[4] https://cba.mit.edu/events/22.08.OM

[5] M. Erni, A. John Hart, D. Trumper, C.E. Owens, A low-cost, open-source cylindrical Couette rheometer. Sci. Rep. **14**(1), 1–15 (2024) 39632889 10.1038/s41598-024-76494-8 PMC11618454

[6] https://www.open-raman.org

[7] U. Gaudenz, 3DFiberSpectrometer. *GaudiLabs*. (2022), https://www.gaudi.ch/GaudiLabs/?page_id=825 and https://github.com/GaudiLabs/3DFiberSpectrometer

[8] https://openflexure.org

[9] H.S. Liao, I. Akhtar, C. Werner, R. Slipets, J. Pereda, J.-H. Wang, E. Raun, L.O. Nørgaard, F.E. Dons, E.E.T. Hwu, Open-source controller for low-cost and high-speed atomic force microscopy imaging of skin corneocyte nanotextures. HardwareX **12**, e00341 (2022) 35936941 10.1016/j.ohx.2022.e00341 PMC9352456

[10] J. de Vos, L. Kincheloe, H. Motza, Plastic Scanner, GitHub https://github.com/Plastic-Scanner

[11] A. Straller, B. Gessler, Identification of plastic types using discrete near infrared reflectance spectroscopy.

[12] J. Vasquez, N. Peek, N. Gershenfeld, Jubilee: an extensible machine for multi-tool fabrication. *Proceedings of the 2020 CHI Conference on Human Factors in Computing Systems*, 2020, pp. 1–12

[13] M. Politi et al., A high-throughput workflow for the synthesis of CdSe nanocrystals using a sonochemical materials acceleration platform. Digit. Discov. **2**(4), 1042–1057 (2023)

[14] U. Gaudenz, PocketPCR. *GaudiLabs*. (2023), https://gaudi.ch/PocketPCR/ and https://github.com/GaudiLabs/PocketPCR

[15] M. Omer et al., Designing for replicability: a qualitative empirical study on the replication of open-source machine tools. Des. Sci. **10**, e30 (2024)

[16] J. Dyvik, Fabricatable machines. *Fellesverkstedet*, (2019), https://github.com/fellesverkstedet/fabricatable-machines/wiki

[17] F.H. Fossdal et al., Fabricatable machines: a toolkit for building digital fabrication machines. *Proceedings of the Fourteenth International Conference on Tangible, Embedded, and Embodied Interaction*, 2020, pp. 1–8

[18] https://gitlab.cba.mit.edu/jakeread/displacementexercise

[19] N. Eghbal, *Working in Public: The Making and Maintenance of Open Source Software* (Stripe Press, San Francisco, 2020)

[20] Y. Benkler, Coase’s Penguin, or, Linux and the nature of the firm. Yale Law J. (2002). 10.2307/1562247

[21] J.R. Read et al., Modular-things: plug-and-play with virtualized hardware. *Extended Abstracts of the 2023 CHI Conference on Human Factors in Computing Systems*. 2023

[22] J.R. Read, N. Peek, N. Gershenfeld, Maxl: distributed trajectories for modular motion. *Proceedings of the 8th ACM Symposium on Computational Fabrication*. 2023

[23] M. Raissi, P. Perdikaris, G.E. Karniadakis, Physics-informed neural networks: a deep learning framework for solving forward and inverse problems involving nonlinear partial differential equations. J. Comput. Phys. **378**, 686–707 (2019). 10.1016/j.jcp.2018.10.045

[24] E. Strand, F. Tourlomousis, N. Gershenfeld, Mesoscale material modeling with memoryless isotropic point particles. J. Comput. Sci. **75**, 102198 (2024). 10.1016/j.jocs.2023.102198

[25] S.P. Johnston, D.O. Kazmer, R.X. Gao, Online simulation-based process control for injection molding. Polym. Eng. Sci. **49**(12), 2482–2491 (2009)

[26] J. Andersson, J. Åkesson, M. Diehl, CasADi: a symbolic package for automatic differentiation and optimal control, in *Recent Advances in Algorithmic Differentiation*. (Springer, Berlin, 2012)

[27] R. Verschueren et al., acados—a modular open-source framework for fast embedded optimal control. Math. Program. Comput. **14**(1), 147–183 (2022)

[28] J. Bradbury et al., JAX: composable transformations of Python+NumPy programs, (2018), http://github.com/jax-ml/jax

[29] T.J. Coogan, D.O. Kazmer, In-line rheological monitoring of fused deposition modeling. J. Rheol. **63**(1), 141–155 (2019)

[30] J.R. Read et al., Online measurement for parameter discovery in fused filament fabrication. Integr. Mater. Manuf. Innov. **13**(2), 541–554 (2024) 39668907 10.1007/s40192-024-00350-w PMC11636983

